# Drought rapidly diminishes the large net CO_2_ uptake in 2011 over semi-arid Australia

**DOI:** 10.1038/srep37747

**Published:** 2016-11-25

**Authors:** Xuanlong Ma, Alfredo Huete, James Cleverly, Derek Eamus, Frédéric Chevallier, Joanna Joiner, Benjamin Poulter, Yongguang Zhang, Luis Guanter, Wayne Meyer, Zunyi Xie, Guillermo Ponce-Campos

**Affiliations:** 1 Climate Change Cluster, University of Technology Sydney, Broadway, New South Wales, 2007, Australia; 2 School of Life Sciences, University of Technology Sydney, Broadway, New South Wales, 2007, Australia; 3 Laboratoire des Sciences du Climat et de l’Environnement, CEA/CNRS/UVSQ, Gif-sur-Yvette, France; 4 NASA Goddard Space Flight Centre, Laboratory for Atmospheric Chemistry and Dynamics, Greenbelt, Maryland, 20771, United States; 5 Institute on Ecosystems and Department of Ecology, Montana State University, Bozeman, Montana, 59717, United States; 6 Jiangsu Provincial Key Laboratory of Geographic Information Science and Technology, International Institute for Earth System Sciences, Nanjing University, Nanjing, Jiangsu, 210023, China; 7 Jiangsu Center for Collaborative Innovation in Geographical Information Resource Development and Application, Nanjing, Jiangsu, 210023, China; 8 Helmholtz Centre Potsdam, GFZ German Research Centre for Geosciences, Potsdam, 14473, Germany; 9 Environment Institute, Ecology and Environment Science, University of Adelaide, South Australia, 5005, Australia; 10 USDA Agricultural Research Service, Southwest Watershed Research Centre, Tucson, Arizona, 85719, United States

## Abstract

Each year, terrestrial ecosystems absorb more than a quarter of the anthropogenic carbon emissions, termed as land carbon sink. An exceptionally large land carbon sink anomaly was recorded in 2011, of which more than half was attributed to Australia. However, the persistence and spatially attribution of this carbon sink remain largely unknown. Here we conducted an observation-based study to characterize the Australian land carbon sink through the novel coupling of satellite retrievals of atmospheric CO_2_ and photosynthesis and *in-situ* flux tower measures. We show the 2010–11 carbon sink was primarily ascribed to savannas and grasslands. When all biomes were normalized by rainfall, shrublands however, were most efficient in absorbing carbon. We found the 2010–11 net CO_2_ uptake was highly transient with rapid dissipation through drought. The size of the 2010–11 carbon sink over Australia (0.97 Pg) was reduced to 0.48 Pg in 2011–12, and was nearly eliminated in 2012–13 (0.08 Pg). We further report evidence of an earlier 2000–01 large net CO_2_ uptake, demonstrating a repetitive nature of this land carbon sink. Given a significant increasing trend in extreme wet year precipitation over Australia, we suggest that carbon sink episodes will exert greater future impacts on global carbon cycle.

Since the beginning of the industrial age, human activities (fossil fuel combustion, land use change, etc.) have driven the atmospheric CO_2_ concentration from about 280 parts per million (ppm) in around 1780 to over 400 ppm in 2015^1^,^2^. The burning of fossil fuels and other human activities are currently adding more than 36 billion metric tons of CO_2_ to the atmosphere each year^3^, producing an unprecedented build-up of this important greenhouse-forcing agent. Each year, terrestrial ecosystems sequester on average about a quarter of fossil fuel emissions and help mitigate global warming^1^,^2^,^3^,^4^,^5^. However, the nature, geographic distribution of land carbon sinks, and how their efficiencies change from year to year are not adequately understood, precluding an accurate prediction of their responses to future climate change and subsequent influences on climate through carbon cycle-climate feedbacks^6^,^7^. Recent evidence suggests that global semi-arid ecosystems provide an important contribution to the global land carbon sink and can dominate inter-annual variability and the trend of global terrestrial carbon cycle^8^,^9^.

Previous studies of the semi-arid carbon sink primarily relied on model outputs^8^,^9^. The results can be subject to uncertainties in input variables such as the assimilation of datasets that are sparse in many regions of the world, and may be further confounded by model assumptions, as suggested by a previous study finding that models can differ substantially in predicting inter-annual variability of the terrestrial carbon cycle^10^,^11^. More importantly, these studies do not fully utilise ground measurements of carbon fluxes (e.g., from eddy-covariance flux towers). An observation-based study of the spatial attribution and temporal evolution of extreme wet-year driven carbon sinks over semi-arid regions has yet to be conducted to date.

Amplification of the hydrological cycle as a consequence of global warming is predicted to increase the frequency and severity of drought in the future^12^,^13^. These extreme drought events, if coupled with warmer temperatures, are expected to profoundly affect ecosystem function and structure, further exerting major impacts on the global carbon cycle and feedbacks to alter the rate of climate change^13^,^14^,^15^,^16^,^17^,^18^,^19^,^20^. Globally, a reduction in terrestrial primary productivity from 2000 to 2009 has been recorded primarily due to drought impact, which weakened the terrestrial carbon sink^17^. Regionally, the 2003 European warm-drought caused broad-scale declines in ecosystem productivity, led to a reverse of 4 years of carbon uptake^21^. The 2000–2004 drought reduced regional carbon uptake over western North America substantially^22^, and also caused large-scale vegetation die-off 23,24,25, further releasing carbon stocks from biomass back to the atmosphere.

Global semi-arid ecosystems are mostly dominated by grasslands, shrublands, and savannas^26^,^27^,^28^. A combination of high biomass turnover rates and their presence in water-limited environments renders semi-arid ecosystems particularly sensitive to drought and wet events^28^,^29^,^30^,^31^,^32^. A recent study using flux tower measurements over semi-arid grasslands in southwest United States identified a precipitation threshold that a semi-arid system could switch from a net sink or a net source of carbon from year to year^32^. Meanwhile, a common temporal and spatial sensitivity of gross CO_2_ uptake to water-availability over a broad diversity of semi-arid ecosystems in North America has also been reported^33^, indicating that fast ecophysiological responses are useful for predicting semi-arid carbon sink/source dynamics under future climatic water availability^34^. Global semi-arid ecosystems are currently threatened by increasing aridity and enhanced warming^35^,^36^, posing concerns on their sustainable ability to absorb carbon, maintain biodiversity, and support human livelihood. A better knowledge of the intrinsic link between hydroclimatic variations and carbon sink-source dynamics over global semi-arid regions, especially those within the Southern Hemisphere which have dominated the recent global land carbon sink anomaly^8^,^9^, is thus urgently needed. To-date, questions such as which ecosystems contributed most to the 2011 land carbon sink anomaly and how they respond when subsequently subjected to drought, remain largely unanswered. Carbon sink allocation into labile or more stable carbon reservoirs and the factors that govern source-sink sensitivity in semi-arid regions must be understood for prediction of long-term global carbon cycle-climate feedbacks.

Here, we assess the spatial allocation and temporal evolution of the land carbon sink over Australia in response to early 21^st^-century hydroclimatic variations. Australia, the driest inhabited continent in the world, is dominated by savannas, grasslands, and shrublands (Supplementary Fig. S1). We focus on Australia not only because of its recent impact on the global carbon cycle^8^,^9^, but also because it experiences the largest climate variability among the continents (Supplementary Fig. S2), thus it is of interest to know how ecosystems behave under such extreme climate variability. Specifically, we want to determine: (1) the detailed spatial allocation of the large net CO_2_ uptake and variations in intrinsic efficiency of using rainfall for taking up carbon during abnormally wet periods; (2) the persistence of the large semi-arid net CO_2_ uptake throughout following dry period; and (3) the recurrence of Australia’s large net CO_2_ uptake in other wet years.

To answer these questions, we employed a multiple observation-based, interdisciplinary approach. To provide a “top-down” atmospheric view of the carbon dynamics, we used inverted net ecosystem carbon production (NEP) derived from satellite retrievals of CO_2_ concentration from Japanese Greenhouse Gases Observing Satellite (GOSAT). We also used remote sensing of vegetation photosynthetic activity, including enhanced vegetation index (EVI) from the Moderate Resolution Imaging Spectroradiometer (MODIS) onboard NASA’s Terra satellite and solar-induced chlorophyll fluorescence (SIF) from the Global Ozone Monitoring Experiment-2 (GOME-2) sensor onboard EUMETSAT’s MetOp-A/B satellites. The EVI and SIF were used in a complementary manner, as SIF is emission-based and directly measures photosynthetic activity, while MODIS EVI is at a higher spatial resolution and enables the direct comparison with *in-situ* micrometeorological flux tower measurements. To characterize the hydroclimatic variations, we used the total water storage change (TWSC) from NASA’s Gravity Recovery and Climate Experiment (GRACE) satellites and standardized precipitation-evapotranspiration drought index (SPEI) computed from long-term ground meteorological datasets. For SPEI, we used data computed at 6-month time scale considering the fact that vegetation across Australia primarily respond to drought at time-scales around 6-months or less. Lastly, as the most direct measure of ecosystem-atmosphere carbon exchange, we used NEP measured by *in-situ* eddy-covariance flux towers from two semi-arid ecosystems in Australia. Furthermore, to infer the contribution from fire emission to the semi-arid carbon-source dynamics, we employed the fire emission data from the fourth generation global fire emission database (GFED4.1s). A combination of multiple observation-based approaches will provide us the most direct and convergent evidence of the detailed spatial attribution, propensity, and the fate of the semi-arid carbon sink.

For our analyses, to accommodate the seasonal rainfall distribution over Australia, we used a ‘hydrological year’ instead of using a calendar year. The hydrological year is defined as from September to next August based on mean monthly rainfall climatology, such that precipitation falling within a hydrological year was mainly used within that year for vegetation productivity. In following text, a hydrological year such as 2010–11 represents a 12-months period from September-2010 to August-2011.

## Results and Discussion

**Figure 1:**
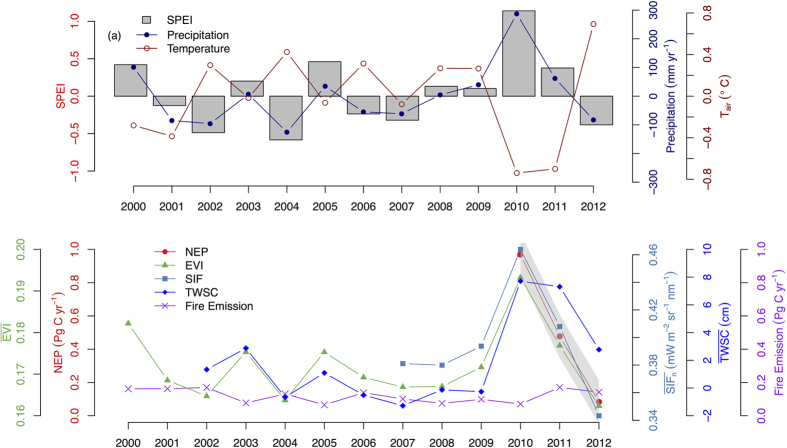
Inter-annual variation in climate, carbon flux and terrestrial primary productivity of Australia from 2000–01 to 2012–13. On the x-axis, 2000 represents the hydrological year starting from September-2000 to August-2011, and so on. (**a**) Variation in continental average annual Standardized Precipitation-Evapotranspiration drought Index (SPEI), anomaly of annual precipitation (mm yr^−1^), anomaly of annual mean daily temperature (T_air_, °C) over Australia from 2000–01 to 2012–13. Anomalies of precipitation and temperature were computed with reference to the 1970–2013 base period. Positive SPEI indicates wetter than the historical median; negative values, drier than median; (**b**) Variation in continental summation of Net Ecosystem Production (NEP) (Pg C yr^−1^), continental average of annual integrated Enhanced Vegetation Index (EVI), continental average of Solar-Induced chlorophyll Fluorescence (SIF_n_, PAR normalized SIF, mW m^−2^ sr^−1^ nm^−1^), continental average of Total Water Storage Change (TWSC, cm), and continental total fire carbon emission (Pg C yr^−1^). NEP is derived from GOSAT atmospheric inversion modeling. The gray shaded area represents the Bayesian uncertainty range of inverted NEP (±1σ).

Early 21^st^-century hydroclimatic variations spanning Australia were characterised by below average precipitation coupled with anomalously high temperature (Fig. 1a). Annual precipitation was below or near the historical average (1970–2013), while annual average temperature was above or near the historical average throughout nearly the entire 2001–02 to 2008–09 (Fig. 1a). This warm-dry period was enclosed by two wet periods, 2000–01 and 2010–11, with another wet period occurring in 2005–06 (Fig. 1a). Australia’s terrestrial ecosystems were strong carbon sinks in 2010–11 hydrological year (~0.97 Pg C yr^−1^) (Fig. 1b; Supplementary Table S1). This strong land carbon sink is corroborated by independent observations from MODIS EVI and GOME-2 SIF, both showing a much enhanced photosynthetic activity during the 2000–01, and 2010–11 wet episodes (Fig. 1b; Supplementary Table S1). The magnitude of the 2010–11 enhanced land carbon sink as derived from GOSAT inversion in this study, if reported based on calendar year, was 0.77 Pg in 2011, which is very similar with 0.79 Pg C reported by Poulter *et al*.^8^ or 0.86 Pg C from another recent study^37^.

Consistent with the results from recent hydrological studies^38^,^39^, the large land carbon sink anomaly in 2010–11 corresponds with a large surplus of water storage (soil moisture + ground water) as indicated by GRACE TWSC (Fig. 1b). A sharp decline in both precipitation and GRACE TWSC after 2011, resulting in a dramatic reduction in NEP, EVI, and SIF from 2011 to 2013 and demonstrating the systematic link between semi-arid carbon sink-source dynamics and hydroclimatic variations (Fig. 1b). This is consistent with a recent study using Australian land surface model, constrained by multiple observational data sources^40^, which also reported a similar sharp decline in continental NEP after the 2010–11 wet period^41^.

Meanwhile, CO_2_ emissions from fire burning were also enhanced following the 2010–11 productive wet period (Fig. 1b). The total fire emission over Australia, as estimated by GFED4.1s, was 0.07 Pg C in 2010–11, ~42% below 2000–2013 average (Fig. 1b). The fire emission was boosted in 2011–12 (0.17 Pg C), one year followed the land carbon sink year, and was slightly reduced to 0.14 Pg C in 2012–13 (Fig. 1b; Supplementary Table S1). The total fire emission over Australia throughout the 2010–11 to 2012–13 period was 0.38 Pg C, about 25% of the size of total net carbon uptake (1.53 Pg C) over this period (Fig. 1b; Supplementary Table S1). This demonstrates the importance of fire emission as an important loss pathway over semi-arid ecosystems.

As a result of the combined effects from drought-induced reduction of photosynthetic activity and enhanced fire emissions, the percentage contribution of Australian terrestrial ecosystems to the global land carbon sink, as inferred from atmospheric inversion data, declined substantially from ~25% in 2010–11 to ~13% in 2011–12, and further reduced to ~2% in 2012–13. These observation-based results demonstrate that the rapid reversals of semi-arid carbon sink anomalies are important contributors to the inter-annual variability of the global terrestrial carbon cycle, as was shown through previous studies^8^,^9^,^42^.

Meanwhile, our results also indicate the potential ecological resilience of Australia’s dryland ecosystems to altered hydroclimatic conditions, as they were able to sequester large amounts of carbon after protracted dry periods (Fig. 1). The resilience of Australian semi-arid ecosystems to drought may be attributed to the fact that these ecosystems are dominated by perennial, drought tolerant species such as *Acacia spp*. (mulga) and *Triodia spp*. (spinifex), each covers ca. 30–35% of Australian semi-arid land area^43^. These species can maintain foliage and minimal metabolic activity during the dry season or drought periods, largely attributed to their highly drought-adapted hydraulic architecture and their ability to tap deep soil water reserves with their lengthy root systems. Meanwhile, drought-adapted biota in these ecosystems also resulted in asymmetric response of Australia’s dryland ecosystems to rainfall, in which these ecosystems responded more strongly to wet than to dry period^44^. As a result of above potential mechanisms, Australian semi-arid ecosystems were able to contribute to 2011 global land carbon sink anomaly significantly after the protracted early 21^st^-centurary warm-dry period, further demonstrating the resilient nature of Australian semi-arid carbon sink (Fig. 1). Future studies are needed to look at the historical data in order to better understand the ecological resilience of Australia’s dryland ecosystems in response to multiple drought and wet periods.

**Figure 2:**
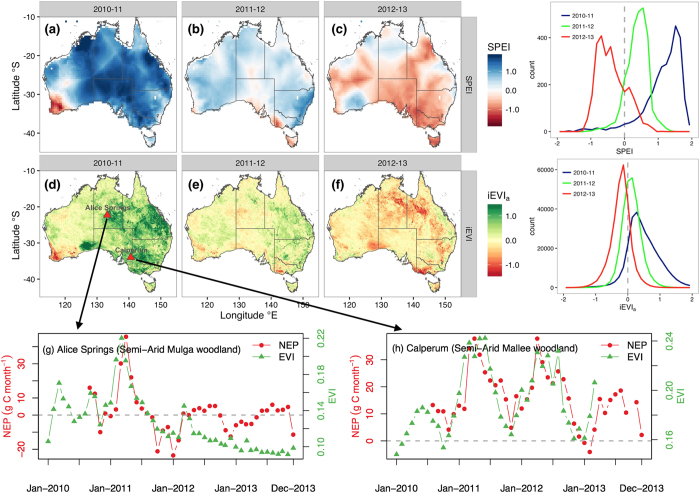
Biogeographic pattern of hydroclimatic condition and terrestrial primary productivity across Australia from 2010–11 to 2012–13. (**a**–**c**) maps of annual average SPEI; (**d**–**f**) maps of annual terrestrial primary productivity anomaly (surrogated by anomaly of annual integrated EVI, or iEVI_a_); (**g**–**h**) MODIS EVI alongside with *in-situ* eddy-covariance tower measured NEP at Alice Springs (semi-arid Mulga woodland) and Calperum (semi-arid Mallee woodland) from Jan-2010 to Dec-2013. Right panels show frequency distribution of SPEI and iEVI_n_ for 2010–11 (blue), 2011–12 (green), and 2012–13 (red) using data from the all of Australia respectively. Maps were drawn using R version 3.1.2 (http://www.r-project.org/).

Carbon exchange over Australia is substantially affected by large-scale contrasting drought and wet conditions (Fig. 2). There was a transition from anomalously wet conditions in 2010–11 (SPEI = 1.14) to anomalously dry condition in 2012–13 (SPEI = −0.38), especially over eastern Australia (Fig. 2a–c; Supplementary Fig. S3). At the same time, the positive anomaly of MODIS EVI in 2011 switched to a negative anomaly in 2012, and by 2013, most areas of Australia exhibited a strong negative MODIS EVI anomaly, suggesting a significant reduction in terrestrial primary productivity and associated weakening of the strength of the carbon sink under drought (Fig. 2d–f; Supplementary Fig. S3).

As the most direct measurement of carbon exchange between an ecosystem and the atmosphere, *in-situ* eddy-covariance (EC) flux tower measurements of NEP at two semi-arid ecosystems were used as ground-truth to verify the patterns in the temporal evolution of carbon fluxes as estimated from the “top-down” methods (Fig. 2g,h). At Alice Springs, the ecosystem was a net sink of carbon in 2011, and rapidly switched to a weak carbon source in subsequent 2012 and 2013 drought years^43^ (Fig. 2g). At Calperum, each year from 2011 to 2013 was a net sink of carbon, although the strength was weakened substantially in 2013 due to drought (Fig. 2h). Most importantly, we found strong agreement between *in-situ* EC flux tower measured NEP and MODIS EVI (Fig. 2g,h), suggesting that temporal variations in carbon fluxes at these semi-arid ecosystems were primarily driven by photosynthetic activity during the carbon sink period. This finding also supports the use of satellite measures of photosynthetic activity (MODIS EVI and GOME-2 SIF) for depicting the spatial allocation of the carbon sink over dryland ecosystems. Meanwhile, we also note the divergence between MODIS EVI and EC flux-tower NEP at Alice Springs during the carbon source period (2012–2013) (Fig. 2g), suggesting that remote sensing measures of photosynthesis is less suitable for identifying the location of carbon emissions during the source period, while more useful for inferring the location of carbon reservoirs during the sink period. The discrepancy between EVI and NEP during the dry period is likely due to: 1) EVI is a measure of photosynthetic capacity, which is more related to net primary productivity (NPP, the difference between photosynthesis and autotrophic respiration); 2) NEP is a balance between photosynthesis and ecosystem respiration. When NEP is primarily driven by variations in photosynthesis, it is expected to see a good correlation between NEP and EVI, as we saw during the land carbon sink year (Fig. 2g). When ecosystem is under drought stress and thus NEP is primarily driven by variations in ecosystem respiration, there should be a divergence between EVI and NEP, as during the period following the land carbon sink year (Fig. 2g).

**Figure 3:**
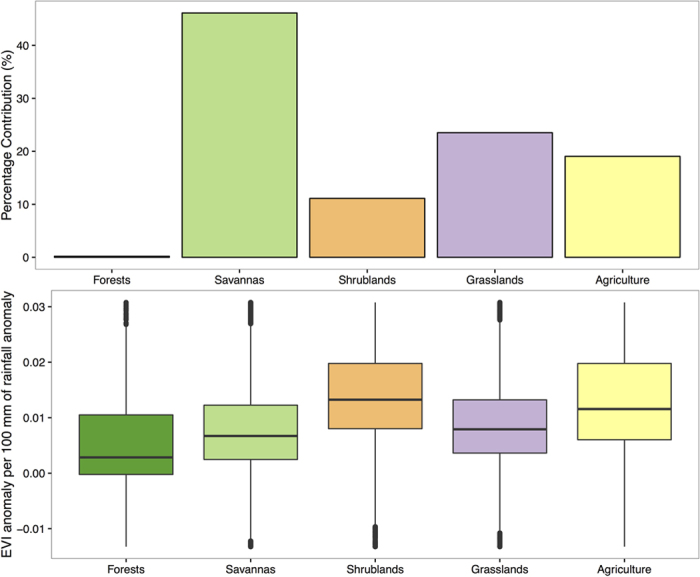
Partitioning of Australia’s terrestrial primary productivity into contributions from major biomes during the 2010–11 carbon sink period. (**a**) Percentage contribution of each biome to Australian 2010–11 productivity anomaly (as indicated by anomaly of EVI). The percent contribution was calculated using MODIS EVI. For each measure, the anomaly of EVI in 2010–11 hydrological year was calculated and then partitioned spatially into different biome type; (**b**) box-plot of EVI anomaly in 2010–11 per 100 mm of rainfall anomaly in 2010–11 generated using all pixels within each biome. This is equivalent to the normalisation of EVI anomaly for each biome by its total area and then further by rainfall anomaly. Thus the ratio is a measure of intrinsic efficiency of an ecosystem in using rainfall for taking up carbon. The upper and lower hinges of the box-plot correspond to the first and third quartiles (the 25th and 75th percentiles) of the data, while the upper and lower whiskers correspond to the maximum and the minimum value of the data. The thick horizontal line within the box represents the median value of the data.

We have shown that the NEP of carbon is primarily driven by photosynthesis during the carbon sink period over Australia’s dryland ecosystems (Fig. 2g,h). We thus inferred the spatial location of the strength of net CO_2_ uptake by partitioning anomaly of MODIS EVI into Australia’s major biomes during the 2010–11 carbon sink period (Fig. 3). Among major biomes, savannas and grasslands had the highest rates of primary productivity anomaly during the 2010–11 La Niña wet periods (Figs 2d and 3a; Supplementary Fig. S1, Table S2). In 2010–11, about 46% of the terrestrial primary productivity anomaly was attributed to savannas and 24% to grasslands (Figs 2d and 3a; Supplementary Fig. S1, Table S2), implying that these two biomes were potentially the biomes with the largest net CO_2_ uptake in Australia during the carbon sink anomaly year, which is in line with another study based on model simulation^45^. The consistence between these independent studies increases the confidence of the spatial partition of the 2010–11 net CO_2_ uptake into different biomes.

To further understand the variation in efficiency of using rainfall for taking up carbon during the 2010–11 carbon sink year, we calculated the ratio between MODIS EVI anomaly and rainfall anomaly for each pixel across Australia during the 2010–11 wet pulse. Our results showed that, despite the fact that savannas and grasslands are potentially the biomes with the largest net CO_2_ uptake in 2010–11, shrublands, which are primarily located over central and southern Australia, have the highest efficiency in using rainfall anomaly for taking up carbon (Fig. 3b; Supplementary Fig. S4, Table S2). Our results indicated that mean rain-use-efficiency of shrublands is significantly higher (*p* < 0.0001, Student’s *t*-Test) than that of grasslands, and also significantly higher (*p* < 0.0001) than that of savannas. Meanwhile, our results also highlighted the large within-biome variation in efficiency of using rainfall for generating productivity anomaly, as indicated by the fact that coefficient of variation (standard deviation/mean) ranges from 60% (shrublands) to 240% (forests) (Supplementary Fig. S4, Table S2). The much larger response of vegetation productivity to very wet year over drier shrublands than wetter biomes such as savannas and forests is likely due to the pulse-response behaviour of the drought-adapted plants in these ecosystems^44^. Besides, we also noticed that for sparsely vegetated areas which have the largest positive asymmetric response of vegetation productivity to wet year^44^, our results showed that part of these sparsely vegetated ecosystems, primarily in Western Australia, did not exhibit a strong productivity response in 2010–11 (Supplementary Fig. S4). The lack of response of these ecosystems to 2010–11 wet year may be attributed to the fact that a persistent decline in GRACE terrestrial water storage (TWS) trend has been observed over Western Australia since 2002^39^. This drying trend as indicated by GRACE TWS imply that vegetation over these regions were stressed during the 2002–2009 period and may have lost resilience, thus unable to respond strongly to the 2010–11 wet year. Future studies, with the incorporation of more field observations, are needed to investigate the exact mechanisms that explain the lack of response of ecosystems over Western Australia to the wet period. These patterns of varying rain-use-efficiency, as derived from observational datasets, are informative and reflect the differences in ecosystem-level carbon-water relationship. Therefore, Earth system models may consider using these patterns as observational test. Specifically, models may test their capability of simulating the spatial nature of the response of terrestrial carbon uptake per unit change in rainfall amount during the extreme wet years, i.e., the sensitivity of terrestrial carbon uptake to precipitation anomaly.

**Figure 4:**
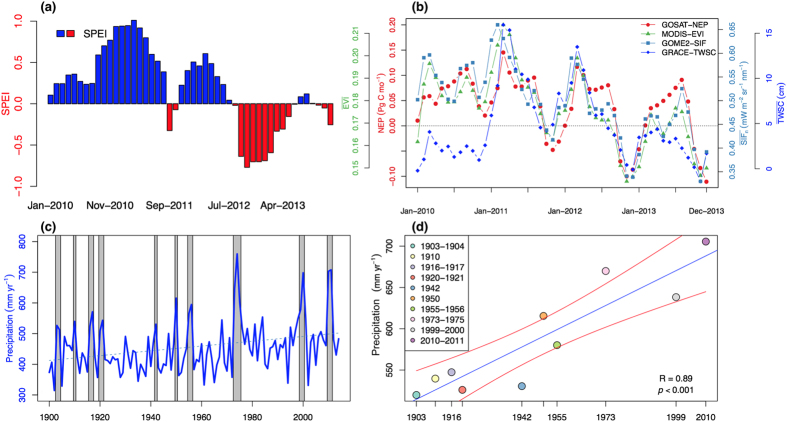
Reduction of net CO_2_ uptake over Australia as determined by subsequent hydroclimatic conditions. (**a**) Monthly SPEI averaged across Australia from 2010 to 2013; (**b**) Variations of monthly NEP, EVI, SIF, and TWSC aggregated across Australia from 2010 to 2013; (**c**) time series of average annual precipitation across Australia from 1900 to 2013. Blue dashed line is the trend line, while grey vertical bars indicate each very wet period since 1900; (**d**) trend in precipitation amount during the very wet periods (e.g., 2010–11). Blue line is linear regression line and red lines indicate 95% confidence intervals. Before calculating the precipitation amount during the very wet periods, the precipitation time series has been linearly detrended.

We have shown that Australia’s ecosystems are strong sinks of CO_2_ during extreme wet periods. An important question that remains is to what extent the strength of the wet year-stimulated net CO_2_ uptake is sustained through non-wet years? Throughout the entire 2010–11 wet period, Australia’s ecosystems remained a large carbon sink, manifested by enhanced photosynthetic activity marked by high MODIS EVI and GOME-2 SIF values (Fig. 4a,b). This active state continued into the first half of 2012 at a much lower rate, owing to reduced water availability as indicated by both SPEI and GRACE TWSC (Fig. 4a,b; Supplementary Fig. S5). Dry conditions prevailed from the late half of 2012 to 2013, resulting in a significant reduction of the net CO_2_ uptake from a strong net sink (−0.77 Pg C in 2010) to a weak sink (−0.12 Pg C in 2013), a 85% reduction in the strength of net CO_2_ uptake (Fig. 4a,b; Supplementary Fig. S5). Our multiple observation-based datasets thus demonstrated the fact that the large semi-arid net CO_2_ uptake originated from extreme La Niña wet period to be very short-lived and a transitory event, in which drought occurrence can rapidly reduce the magnitude of carbon uptake (Fig. 4a,b; Supplementary Fig. S5).

Australia is situated in a region of extreme climate variability, creating several-fold larger fluctuations in precipitation and terrestrial primary productivity than encountered in all other continents (Supplementary Fig. S2). Exceptionally large vegetation productivity was recorded over Australia during both of the recent two La Niña-induced very wet periods (2000–01 and 2010–11) (Fig. 1b), thus demonstrating that the large land carbon sink of Australia reported in 2010–11 was not an isolated and unique event, but rather a potentially episodic occurrence under strong wet phases. This is also reflected by the enhanced fire emissions throughout both the 2000–01 to 2002–03 and 2010–11 to 2012–13 periods (Fig. 1b). Precipitation and hence soil moisture availability are the primary drivers of semi-arid ecosystems carbon dynamics^44^; we therefore expect that the exceptionally large land carbon sink, coupled to very wet phases, will be observed again in the future.

An intriguing and important question is whether the magnitude of Australia’s next carbon sink in a future wet episode will exhibit a similar strength as in 2010–11 or whether it will become stronger or weaker? The historical climatological record for Australia reveals that the precipitation amounts during wet years, such as 2000–01 and 2010–11, have increased significantly since 1900 (increasing by 11.66 mm per year, *p* < 0.05) (Fig. 3c–d; Supplementary Fig. S6). Meanwhile, an overall wetting trend has resulted in the expansion of vegetation cover over Australia since 1981^8^,^46^ (Fig. 4c; Supplementary Fig. S7). Recent study has found that dryland ecosystems in Australia tend to respond more strongly to wet period than drought, i.e., positive asymmetric type of responses, attributable to the pulse-response behaviour of these drought-adapted plants^44^. These findings together suggested that the magnitudes of the Australian semi-arid sink associated with wet-extremes will increase in coming decades. The consequence of this trend will have important implications for global carbon cycling, as shown during the 2011 global land carbon sink anomaly period^8^,^9^.

In summary, multiple independent lines of evidence create a compelling argument that extreme wet year driven, semi-arid land carbon sink events are transient and dissipate rapidly under ensuing drought. The efficiencies of these semi-arid carbon sinks are highly sensitive to hydroclimatic variations and are particularly vulnerable to drought. The spatial attribution of the land carbon sink to tropical savannas and grasslands refines previous attributions to semi-arid ecosystems^7^,^8^. The finding of large variations in rainfall use efficiency among biomes for generating productivity anomaly in 2010–11 provides insight into the intrinsic effectiveness for taking up carbon. The two large carbon sink events, in 2000–01 and 2010–11, were coupled with La Niña-induced wet pulses, and demonstrated an episodic nature of Australia’s land carbon sink. Given a continuation of the increasing trend in extreme wet-year precipitation amounts since 1900 over Australia, we hereby suggest that the large land carbon sink will be observed again with greater magnitude in the future. We conclude that by contributing more positively to the global carbon balance with greater net CO_2_ uptake during the forthcoming more intensive very wet years, Australia’s ecosystems will play a more significant role in the global carbon cycle in coming decades. This study also demonstrated the great potential of integrating satellite and surface observations to provide a big, unifying picture of terrestrial carbon sink-source dynamics over a continental scale. The use of multiple observational data sources (e.g., GOSAT atmospheric CO_2_ sampling, GOME-2 SIF, and GRACE TWSC) increases the robustness of land carbon sink-source analyses that will essentially lead to an improved understanding of the hydroclimatic drivers and pulse response behaviour of carbon fluxes over dryland ecosystems. The rapid dissipation of large semi-arid net CO_2_ uptake under drought as revealed by this study presents important observational tests for Earth System models to accurately depict terrestrial carbon dynamics under intensified hydrological extremes.

## Methods

We use multiple observation-based data sources to diagnose the geographic distribution and temporal evolution of Australia’s land carbon sinks in extremely wet years. Continental-wide biospheric CO_2_ fluxes were inverted using retrievals of atmospheric CO_2_ from Japanese Greenhouse gases Observing SATellite (GOSAT), with the Bayesian uncertainty statistics of the estimated fluxes were defined by a posterior error covariance matrix (see Supplementary information: Atmospheric inversion of biospheric carbon fluxes). Vegetation photosynthetic activity was indicated by two satellite measures: (1) Enhanced Vegetation Index (EVI), which is a composite measure of leaf area, chlorophyll content, and canopy architecture, derived from Moderate Resolution Imaging Spectroradiometer (MODIS) on-board NASA’s Terra satellite (MOD13C1, Collection 5); (2) Solar-Induced chlorophyll-Fluorescence (SIF), which is emitted by the photosystem II of the chlorophyll molecules of assimilating leaves, derived from the Global Ozone Monitoring Experiment-2 (GOME-2) instrument on-board EUMETSAT’s MetOp-A/B satellites (version 26, level-3) (see Supplementary information: Satellite measured vegetation photosynthetic capacity). To determine drought severity, we use the Standardised Precipitation-Evapotranspiration Index (SPEI), which is a multi-scalar drought index that takes into account both precipitation and temperature (see Supplementary information: Standardised Precipitation-Evapotranspiration drought Index). Total water storage change (TWSC) derived from NASA’s Gravity Recovery and Climate Experiment (GRACE) observations was used as an integrative measure of water storage change over continental scale (see Supplementary information: GRACE Terrestrial Total Water Storage Change). We use gridded rain gauge and temperature datasets provided by the National Climate Centre, Australian Bureau of Meteorology (see Supplementary information: Gridded meteorological datasets). Micrometeorological flux tower measured net ecosystem production (NEP) data (Level 3) is provided by OzFLUX network (see Supplementary information: *In-situ* measurements of NEP). To infer the contribution of fire emissions to the sink-source dynamics and further refine our understanding of carbon loss pathway over semi-arid ecosystems, we used fire emission data from the fourth-generation global fire emissions database (GFED4.1s) (see Supplementary information: Global Fire Emissions Database). Major vegetation group map provided by Australian National Vegetation Information System (NVIS, v4.1) was used to derive the contribution of each biome type to continental carbon fluxes (see Supplementary information: Vegetation map). The 26 NVIS vegetation groups were grouped into five major biomes: forest, savanna, shrubland, grassland, and agriculture. The savanna biome, a tree-shrub-grass multi-strata system, includes all open forests, woodlands, as well as parts of shrublands, with both *Eucalyptus*, *Acacia* or other trees as the dominant canopy species. The grassland biome includes hummock grassland, tussock grassland, and other grassland types.

## Additional Information

**How to cite this article**: Ma, X. *et al*. Drought rapidly diminishes the large net CO_2_ uptake in 2011 over semi-arid Australia. *Sci. Rep*. **6**, 37747; doi: 10.1038/srep37747 (2016).

**Publisher's note:** Springer Nature remains neutral with regard to jurisdictional claims in published maps and institutional affiliations.

## Supplementary Material

Supplementary Information
