# Supplementary material for: Drought rapidly diminishes the large net CO2 uptake in 2011 over semi-arid Australia
Source: Sci Rep. 2016 Nov 25;6:37747. doi: 10.1038/srep37747 (PMC5123568; doi:10.1038/srep37747)
Supplement: Supplementary Information [file srep37747-s1.pdf]

Supplementary Information

Drought rapidly diminishes the large net CO_2 uptake in 2011 over semi-arid Australia

Xuanlong Ma[a,*], Alfredo Huete[a], James Cleverly[b], Derek Eamus[b], Frédéric Chevallier[c], Joanna Joiner[d], Benjamin Poulter[e], Yongguang Zhang[f,g], Luis Guanter[h], Wayne Meyer[i], Zunyi Xie[a], and Guillermo Ponce-Campos[j]

[a] Climate Change Cluster, University of Technology Sydney, Broadway, New South Wales, 2007 Australia;

[b] School of Life Sciences, University of Technology Sydney, Broadway, New South Wales, 2007 Australia;

[c] Laboratoire des Sciences du Climat et de l'Environnement, CEA/CNRS/UVSQ, Gif-sur-Yvette, France;

[d] NASA Goddard Space Flight Centre, Laboratory for Atmospheric Chemistry and Dynamics, Greenbelt, Maryland, 20771 United States;

[e] Institute on Ecosystems and Department of Ecology, Montana State University, Bozeman, Montana, 59717 United States;

[f] Jiangsu Provincial Key Laboratory of Geographic Information Science and Technology, International Institute for Earth System Sciences, Nanjing University, Nanjing, Jiangsu, 210023 China;

[g] Jiangsu Center for Collaborative Innovation in Geographical Information Resource Development and Application, Nanjing, Jiangsu, 210023 China;

[h] Helmholtz Centre Potsdam, GFZ German Research Centre for Geosciences, Potsdam, 14473 Germany;

[i] Environment Institute, Ecology and Environment Science, University of Adelaide, South Australia, 5005 Australia;

[j] USDA Agricultural Research Service, Southwest Watershed Research Centre, Tucson, Arizona, 85719 United States

* Author to whom correspondence should be addressed X.M. (email: xuanlong.ma@uts.edu.au).

This supplementary information file contains:

1. Supplementary materials and methods
    1.1. *Atmospheric inversion of biospheric carbon fluxes*
    1.2. *Satellite measured vegetation photosynthetic capacity*
    1.3. *In-situ measurements of net ecosystem carbon exchange by eddy-covariance flux towers*
    1.4. *Standardised Precipitation-Evapotranspiration drought Index*
    1.5. *GRACE Terrestrial Total Water Storage Change*
    1.6. *Gridded meteorological datasets*
    1.7. *Global fire emissions database*
    1.8. *Identifying precipitation pulses*
    1.9. *Vegetation map*
2. Supplementary table and figures
3. Supplementary references

1. Supplementary materials and methods

1.1 Atmospheric inversion of biospheric carbon fluxes

Atmospheric CO_2 inversions estimate surface carbon fluxes from an "atmospheric point of view" (i.e., "top-down"), using an atmospheric transport model by optimally fitting, in a statistical sense, the high-fidelity atmospheric measurements of CO_2 concentration sampled from a global network of more than 100 sites and prior information about the carbon fluxes[1]. To improve the density of atmospheric CO_2 measurements, remote sensing techniques have been developed for retrieving column-averaged dry air CO_2 mole fractions (χCO_2) from satellite radiance measurements[2-3]. The Thermal And Near-infrared Sensor for carbon Observation-Fourier transform spectrometer (TANSO-FTS) onboard the Japanese Greenhouse Gas Observing Satellite (GOSAT) is the first instrument that was designed to provide the accuracy, resolution, and coverage that are sufficient to characterize the variability of regional sources and sinks of CO_2 on seasonal and inter-annual time

scales[4]. The use of such data is still in its learning phase[5], but Australia is a part of the globe where GOSAT inversion results appear to be robust[6]. In this study we use CO_2 surface fluxes inferred from NASA's Atmospheric CO_2 Observations from Space project retrievals, build 3.5 (ACOS B3.5). The inversion scheme is the global one from the Monitoring Atmospheric Composition and Climate service (MACC, www.copernicus-atmosphere.org)[7]. The MACC inversion scheme is based on an LMDZ (Laboratoire de Météorologie Dynamique) atmospheric transport model[8], while the prior fluxes are estimated using the ORCHIDEE (Organising Carbon and Hydrology In Dynamic Ecosystems) land surface model[9], and fire emission is obtained from Global Fire Emissions Database (GFED) version 4.1[10]. Uncertainty of the inverted CO_2 surface fluxes is estimated using the Bayesian error statistics defined by a posterior error covariance matrix. This matrix has been computed from a stochastic ensemble of inversions that is consistent with the assigned prior error statistics and with the assigned observation error statistics[11].

1.2 Satellite measured vegetation photosynthetic capacity

Approximately 14 years (February 2000 - December 2013) of 16-day 0.05° resolution MODIS Enhanced Vegetation Index (EVI) data (MOD13C1, Collection 5) were obtained from the NASA/USGS data archive centre (https://lpdaac.usgs.gov/). Quality control was applied to retain high-quality observations while minimising aerosol and cloud contamination. EVI is a proxy for canopy “greenness”, which is an integrative composite property of green foliage, leaf chlorophyll content, and canopy architecture[12]. Annual integrated EVI (termed iEVI) has been widely used as a remote sensing surrogate of annual vegetation productivity from arid grassland to forests. In Australia, MODIS EVI has been found to be strongly correlated with eddy-covariance flux tower derived gross primary productivity (GPP)[13]. As an independent and more direct measure of photosynthesis, we use version 26 global monthly (level 3) solar-induced chlorophyll fluorescence (SIF) at ground footprint of 0.5°×0.5° from Sep-2007 to Dec-2014 retrieved from the Global Ozone Monitoring Experiment 2 (GOME-2) instrument. SIF is emitted by photosynthetically active chlorophyll molecules: part of the energy absorbed by chlorophyll is not used for carbon fixations, but re-emitted at longer wavelengths in the 650-800 nm spectral region[14]. SIF measurements from space offer an alternative means for assessing terrestrial photosynthetic activity and productivity at regional to global scales[15-17]. Despite its coarse-resolution (50km) at this moment, the GOME-2 SIF provides a complementary indicator of photosynthetic rate

of traditional spectral vegetation indices because SIF is emission instead of reflectance, thus avoids the contaminations from soil background[16-18]. To minimise the latitudinal and temporal variations in fluorescence retrievals due to the variations in incoming solar radiation, the SIF was normalised by the cosine of the solar zenith angle (surrogate of top-of-atmosphere solar radiation)[15]. The seasonality of SIF is strongly correlated with that of GPP derived from flux tower measurements[17-18]. It has been shown that negative SIF anomalies, particularly for croplands and grasslands, is related to drought-induced reduction in photosynthetic light-use-efficiency[19].

1.3 In-situ measurements of net ecosystem carbon exchange by eddy-covariance flux towers

Eddy-covariance (EC) flux towers provide the most direct measurements of carbon fluxes between an ecosystem and the atmosphere. A full 2 years and 3 months continuous record of net ecosystem production (NEP) from Sep-2010 to Dec-2013 measured by two EC flux towers located at Australia's unique semi-arid ecosystems (refer Fig. 1g for locations of these two sites) were accessed from the OzFLUX network (http://data.ozflux.org.au/portal/home.jspx). The Alice Springs site is located at central Australia semi-arid Mulga woodland (MAP = 443±222 mm, 133.25°E 22.28°S)[20-21], while the Calperum site is located at the southeast Australia semi-arid Mallee woodland (MAP = 251±157 mm, 140.59°E 34.00°S)[22]. Original 30-min Level 3 data was aggregated to monthly and annual values for each site.

1.4 Standardised Precipitation-Evapotranspiration drought Index

To characterise the extent and severity of drought, we used the global 0.5° gridded monthly Standardised Precipitation-Evapotranspiration Index (SPEI) provided by digital CSIC (Institutional Repository of the Spanish National Research Council, http://sac.csic.es/spei/database.html)[23]. SPEI takes into account both precipitation and temperature to determine drought severity and reflects the cumulative effect of the imbalance between atmospheric supply (precipitation) and demand (reference evapotranspiration). Positive SPEI indicates water-balance greater than historical median, and negative SPEI indicates water-balance less than historical median. Because the SPEI is normalised, wetter and drier climates can be represented in the same scale. The SPEI is calculated using monthly meteorological dataset from the Climatic Research Unit of the University of East Anglia

(CRU-TS, version 3.22) in which reference evapotranspiration was estimated using the FAO-56 Penman-Monteith equation.

1.5 GRACE Terrestrial Total Water Storage Change

Global terrestrial total water storage change (TWSC) derived from Release-5 Level-2 Gravity Recovery and Climate Experiment (GRACE) data was obtained from NASA's Jet Propulsion Laboratory (http://grace.jpl.nasa.gov/data/get-data/). The GRACE mission was launched by NASA and the German Aerospace Centre (DLR) in March 2002. The system consists of twin satellites, one following the other at a distance of ~220 km, in identical Earth orbits. GRACE was designed to exploit the unique relationship between variations in the gravity field and changes in mass at the Earth's surface[24]. Because changes in gravity on land are mainly caused by changes in water-storage, variations of GRACE-derived total water storage (TWS) can be obtained by subtracting the monthly data from the historical mean, thus representing total water storage change (TWSC) at any given month relative to the 2004-2009 baseline mean. TWSC is a good measure of vertically integrated changes in groundwater, surface water, soil moisture, snow water and biological water. For this study, an ensemble average TWSC was calculated using GRACE data processed independently by three research centres (NASA's JPL, University of Texas Center for Space Research, and Geo-Forschungs-Zentrum Potsdam). The GRACE TWSC data from July 2002 to December 2014 is gridded at monthly and 1° resolution, with units of cm.

1.6 Gridded meteorological datasets

We used high-resolution (5 km × 5 km) gridded precipitation dataset developed for the Australian Water Availability Project (AWAP)[25]. This dataset uses topography-resolving analysis methods applied to all available monthly precipitation data that pass a series of internal quality control tests. The temperature time series is calculated from a homogeneous temperature dataset (Australian Climate Observations Reference Network-Surface Air Temperature, or ACORN-SAT), which is developed for monitoring climate variability and change in Australia[25]. For global analysis, we used the global monthly 0.25° precipitation dataset provided by NOAA's Global Precipitation Climatology Centre (GPCC, version 6)[26].

1.7 Global fire emissions database (GFED)

We used the latest fourth-generation gridded (0.25°) monthly global fire emissions database (GFED4.1s) (http://www.globalfiredata.org)[10, 27-28]. Fire emissions were estimated by assimilation of satellite observations on fire activity and vegetation productivity from multiple spaceborne sensors, including MODIS burned area maps, active fire information from the Tropical Rainfall Measuring Mission (TRMM) Visible and Infrared Scanner (VIRS) and the Along-Track Scanning Radiometer (ATSR) sensors[27]. The primary update of GFED V4.1s as compared to previous version is the consideration of small fire burned area, as described in Randerson et al.[28]. Monthly fire emissions over Australia were spatially aggregated to the entire continent and temporally aggregated to each year from 2000 to 2013. Please refer Supplementary Table S1 for Australia continent-wide total fire emissions for each year.

1.8 Identifying very wet years

To identify the precipitation pulses while taking into account the long-term trend in annual precipitation at any given location, the original time series of annual precipitation was first linearly-detrended (PPT_{dt}). The second step calculated the standard anomaly of detrended annual precipitation (σPPT_{dt}). Then very wet years were identified when an anomaly in detrended annual precipitation for any given location and given year was equal to or greater than σPPT_{dt}. The intensity for each very wet year (mm yr^{-1}) was defined as the annual precipitation for that year. For continuous wet years, e.g., 2010-2011, we believe that these should be treated as a single very wet period since their influences on terrestrial ecosystems were in a continuous rather than discrete manner. Therefore, the intensity of continuous very wet years was calculated as the mean of annual precipitation for the period of continuous very wet years (e.g., mean of 2010 and 2011). The linear trend of precipitation of very wet years from 1900 to 2013 was then calculated for each grid cell across the entity of Australia. *Supplementary Fig. S6* shows a graphical illustration of the steps for identifying very wet years. Significant tests were applied and only linear trends with $p < 0.05$ are retained for further analysis.

1.9 Vegetation map

We used the Australian Major Vegetation Group (MVG) dataset provided by National Vegetation Information System (NVIS, v4.1)[29]. To understand biome-level contribution to carbon sink anomaly, we grouped 26 NVIS major vegetation groups into five biomes: forest,

savanna, shrubland, grassland, and agriculture. The forest biome includes rainforest and closed forests. The savanna biome, the tree-shrub-grass multi-strata system, includes all open forests, woodlands, as well as parts of shrublands, with both *Eucalyptus*, *Acacia* or other trees as dominant canopy species[30]. The grassland biome includes hummock grassland, tussock grassland, and other grassland types. The agricultural biome includes cropland and pastures.

2. Supplementary table and figures

Supplementary Table S1. Summary of Australia's continental-aggregated annual NEP, precipitation, temperature, SPEI, NEP, EVI, SIF, and fire emission for each hydrological year (September-August) from 2000-01 to 2012-03. Total NEP is computed using all pixels from Australia (7.69 million km^2).

Year	P (mm yr-1)	T (°C)	SPEI	Total NEP (Pg C yr-1)	NEP per unit area (g C m-2 yr-1)	EVI	SIF	Fire Emission (Pg C yr-1)
**2000-01**	606	21.72	0.42			0.18		0.16
**2001-02**	420	21.61	-0.13			0.17		0.16
**2002-03**	409	22.29	-0.49			0.16		0.17
**2003-04**	511	21.98	0.20			0.18		0.08
**2004-05**	379	22.42	-0.58			0.16		0.13
**2005-06**	539	21.93	0.46			0.18		0.07
**2006-07**	450	22.31	-0.23			0.17		0.14
**2007-08**	443	21.92	-0.32			0.17	0.38	0.10
**2008-09**	509	22.26	0.13			0.17	0.38	0.07
**2009-10**	545	22.26	0.10			0.17	0.39	0.10
**2010-11**	792	21.26	1.14	0.97	126.14	0.19	0.46	0.07
**2011-12**	567	21.30	0.38	0.48	62.42	0.18	0.41	0.17
**2012-13**	422	22.69	-0.38	0.08	10.40	0.16	0.34	0.14
**Mean±1 σ**	507±110	22.00±0.43	0.05±0.48	0.51±0.44	66.32±57.97	0.17±0.01	0.39±0.04	0.12±0.04

P: anomaly of annual precipitation relative to the average of 1970-2013 (mm yr^{-1}); T: anomaly of annual average temperature relative to the average of 1970-2013 (°C); SPEI: Standardised Precipitation-Evapotranspiration Index; NEP: Net Ecosystem Production; EVI: annual average MODIS EVI; SIF: annual average GOME-2 SIF; Fire Emission : annual fire carbon emission from GFED.

Supplementary Table S2. Summary of climatology, percentage contribution of each biome to Australia's terrestrial primary productivity anomaly (surrogated by MODIS EVI anomaly), as well as mean rain-use-efficiency (RUE) of each biome during the 2010-2011 La Niña wet period. Mean RUE for each biome was calculated as biome-level average of the ratio between EVI anomaly and rainfall anomaly in 2010-11.

Biome	Area (million km^2)	Percentage Coverage (%)	MAP (mm)	MAT (°C)	Contribution to 2010-11 terrestrial primary productivity (MODIS EVI) (%)	Mean RUE±1σ (EVI per 100 mm of rainfall)
**Forest**	0.05	0.65	1267	16.34	1	0.0030 ± 0.0072
**Savanna**	3.94	51.08	541	22.34	46	0.0074 ± 0.0071
**Shrubland**	0.57	7.60	249	20.62	11	0.0128 ± 0.0082
**Grassland**	1.95	24.83	398	24.36	24	0.0086 ± 0.0074
**Agriculture**	1.16	15.83	573	18.87	19	0.0109 ± 0.0082

MAP: mean annual precipitation; MAT: mean annual temperature.

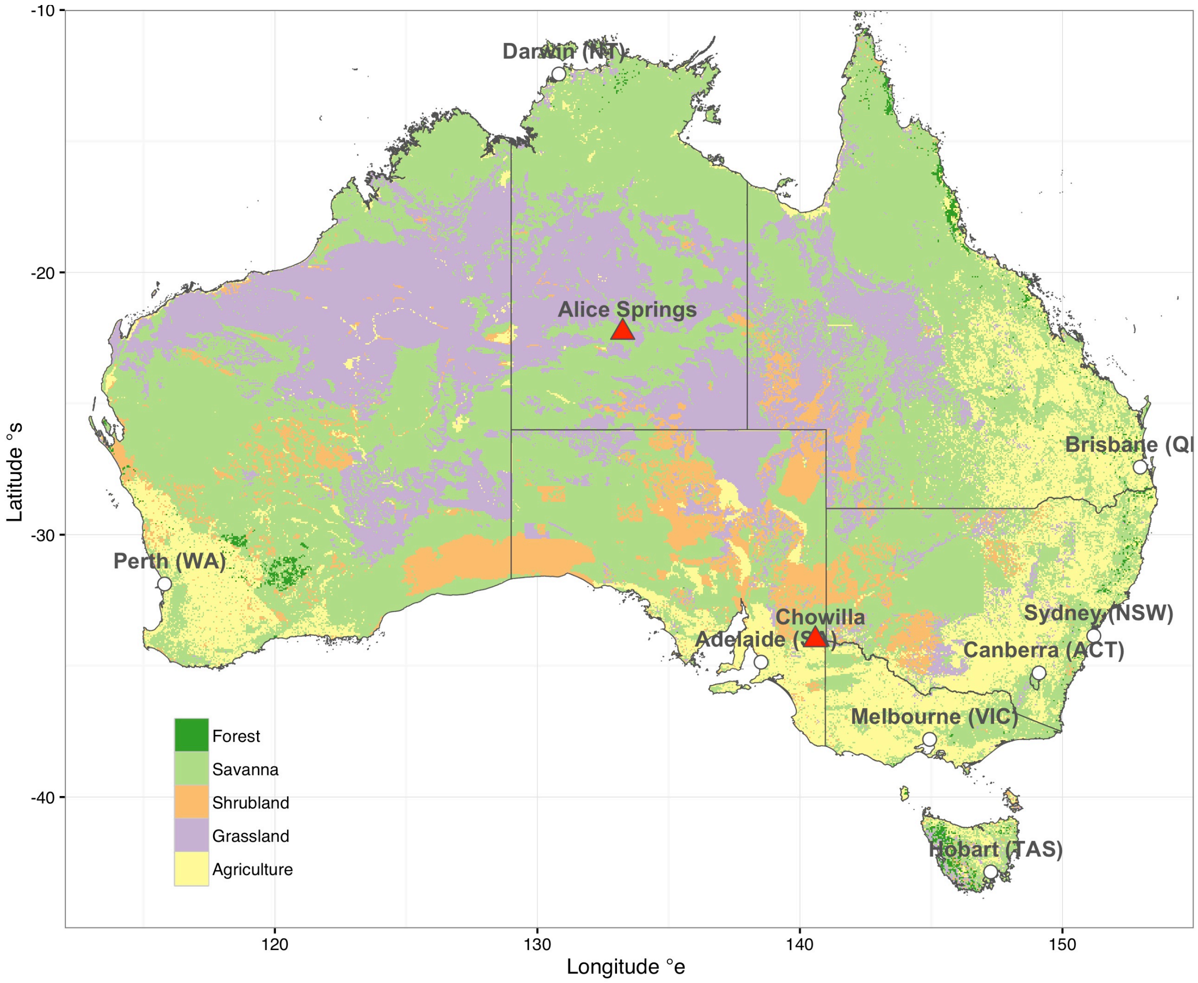

Supplementary Figure S1. Map of Australia’s biome types. Classification of Australia's ecosystems into biome types using Major Vegetation Group map provided by Australian National Vegetation Information System (NVIS, v4.1). Australia’s major cities are labelled as white solid circles, while red solid triangles are two eddy-covariance (EC) flux tower sites located in central and south-east Australia, providing ground measurements of net ecosystem carbon exchange for verifying the ‘top-down’ estimates. Map was drawn using R version 3.1.2 (http://www.r-project.org/).

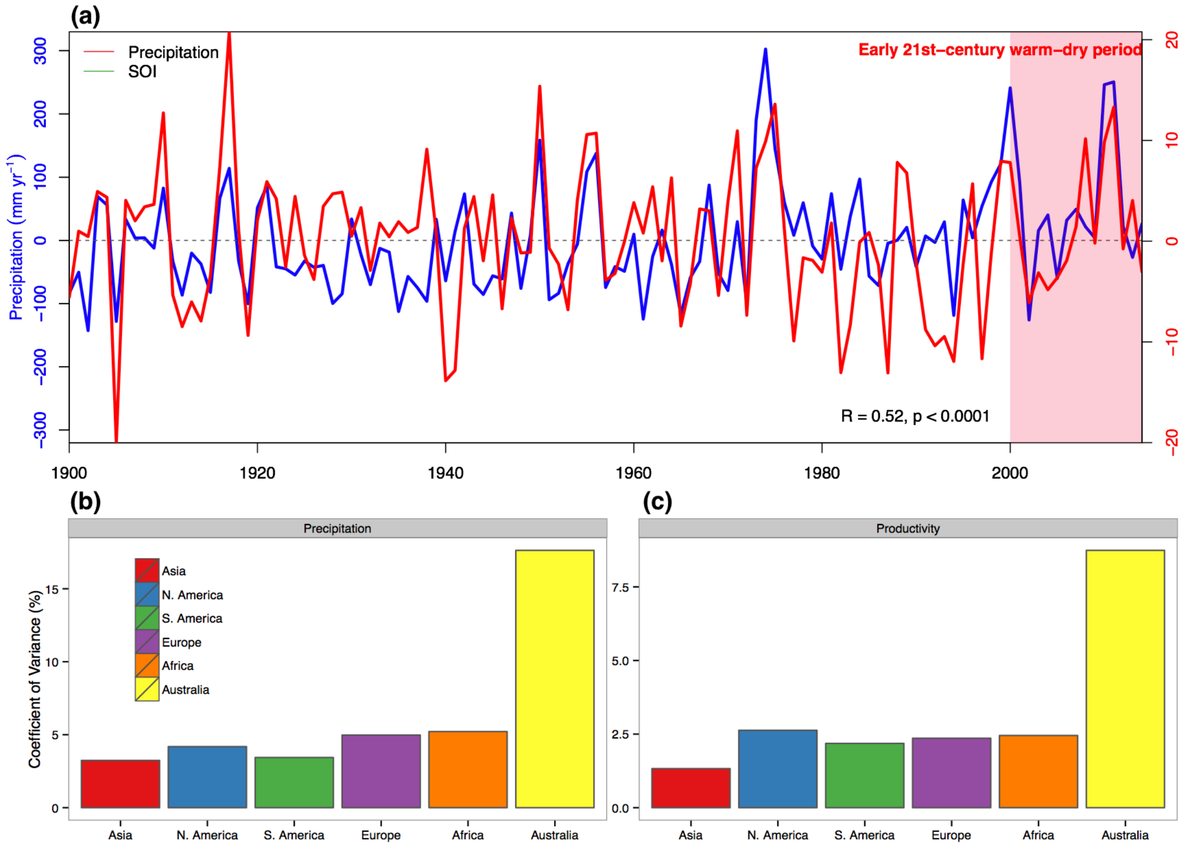

Supplementary Figure S2. Inter-annual variance of Australia's precipitation. (a) Variations in annual precipitation anomaly averaged across the entirety of Australia alongside the Southern Oscillation Index (SOI) from 1900 to 2014; (b) Coefficient of variance (%) in annual precipitation and annual vegetation productivity of six continents. Inter-annual variance is represented by coefficient of variance (CV, %). Global precipitation data is from NOAA Global Precipitation Climatology Centre (GPCC, v6). Yearly integrated MODIS Enhanced Vegetation Index (EVI) is used as a proxy for vegetation productivity. Reference period: 2000-2014. SOI data is obtained from Bureau of Meteorology, Australia.

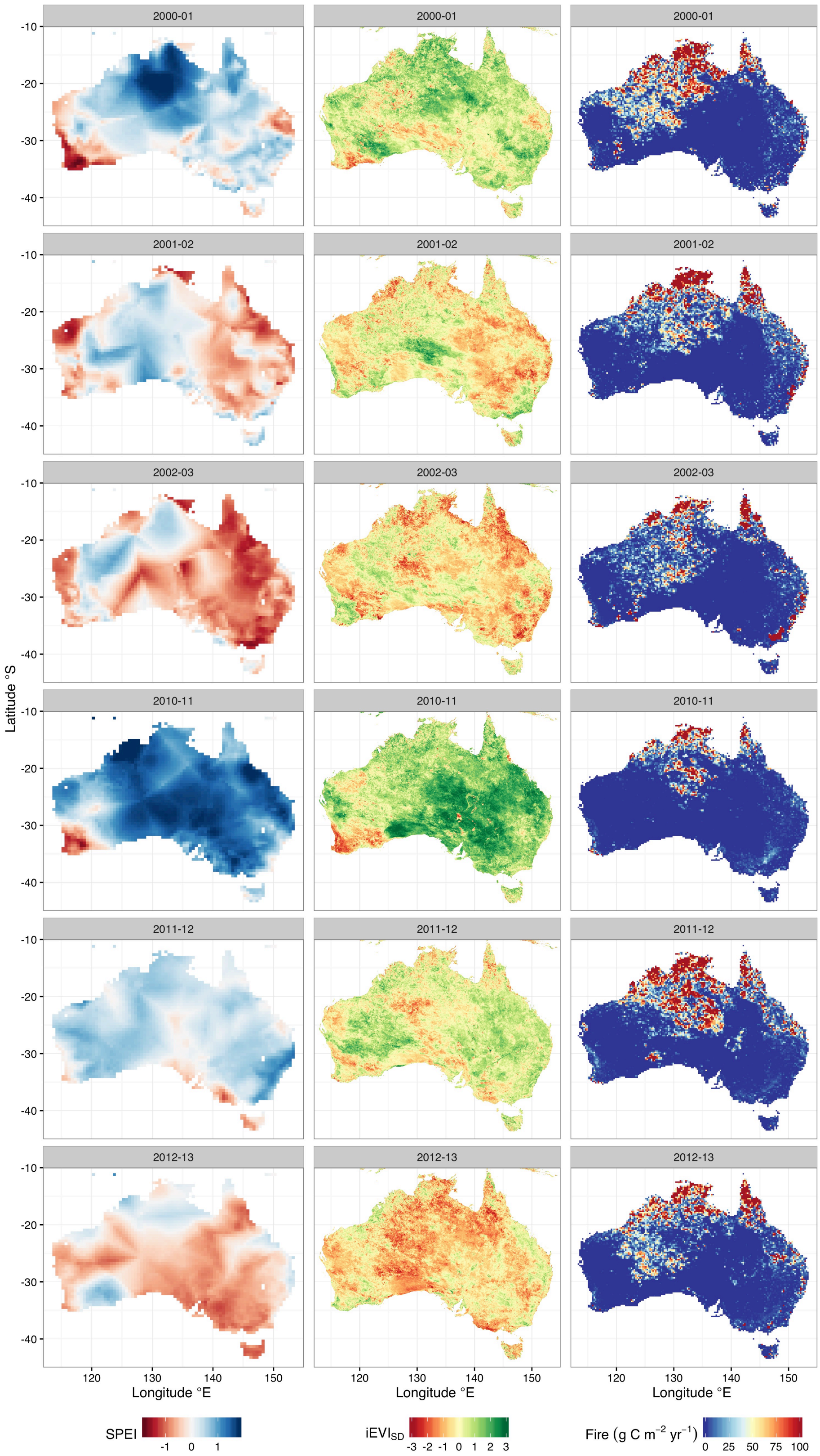

Supplementary Figure S3. Biogeographic patterns in SPEI, iEVI (standardised anomaly) and SIF (standardised anomaly), as well as fire carbon emission over Australia for the 2000-01 to 2002-03 and 2010-11 to 2012-13 time periods respectively. Noted that 2000-01 represents a hydrological year starting from September-2000 to August-2001. Maps were drawn using R version 3.1.2 (http://www.r-project.org/).

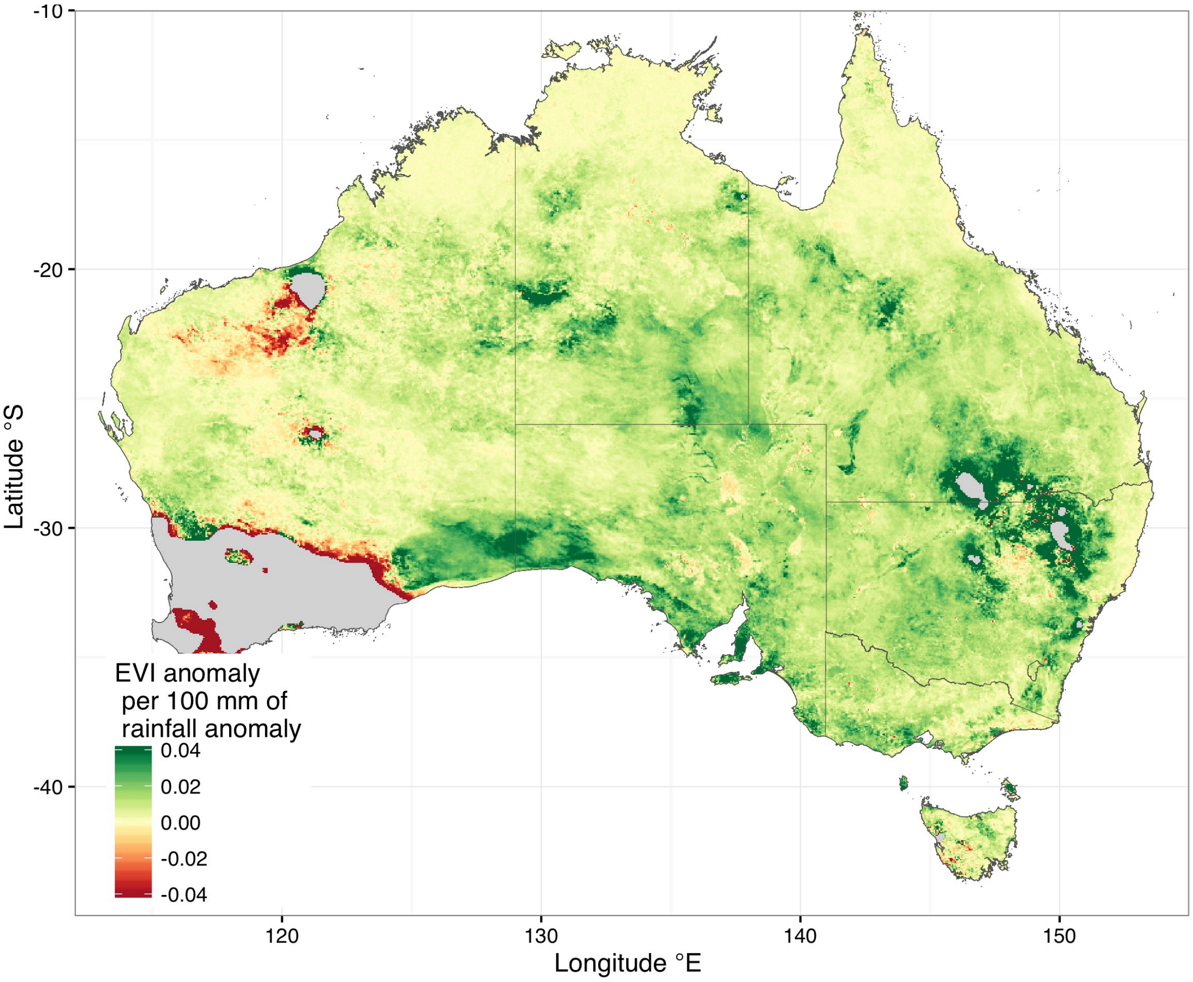

Supplementary Figure S4. Spatial variations in the ratio between EVI anomaly and rainfall anomaly during the 2010-11 La Niña wet period. This map indicates the variations in efficiency of vegetation in using rainfall for generating productivity anomaly across space. Areas with negative rainfall anomaly in 2010-11 were shown in grey. Areas with negative ratio between EVI anomaly and rainfall anomaly indicate ecosystems that showed a decline in vegetation productivity given a wet pulse in 2010-11. Map was drawn using R version

3.1.2 (http://www.r-project.org/).

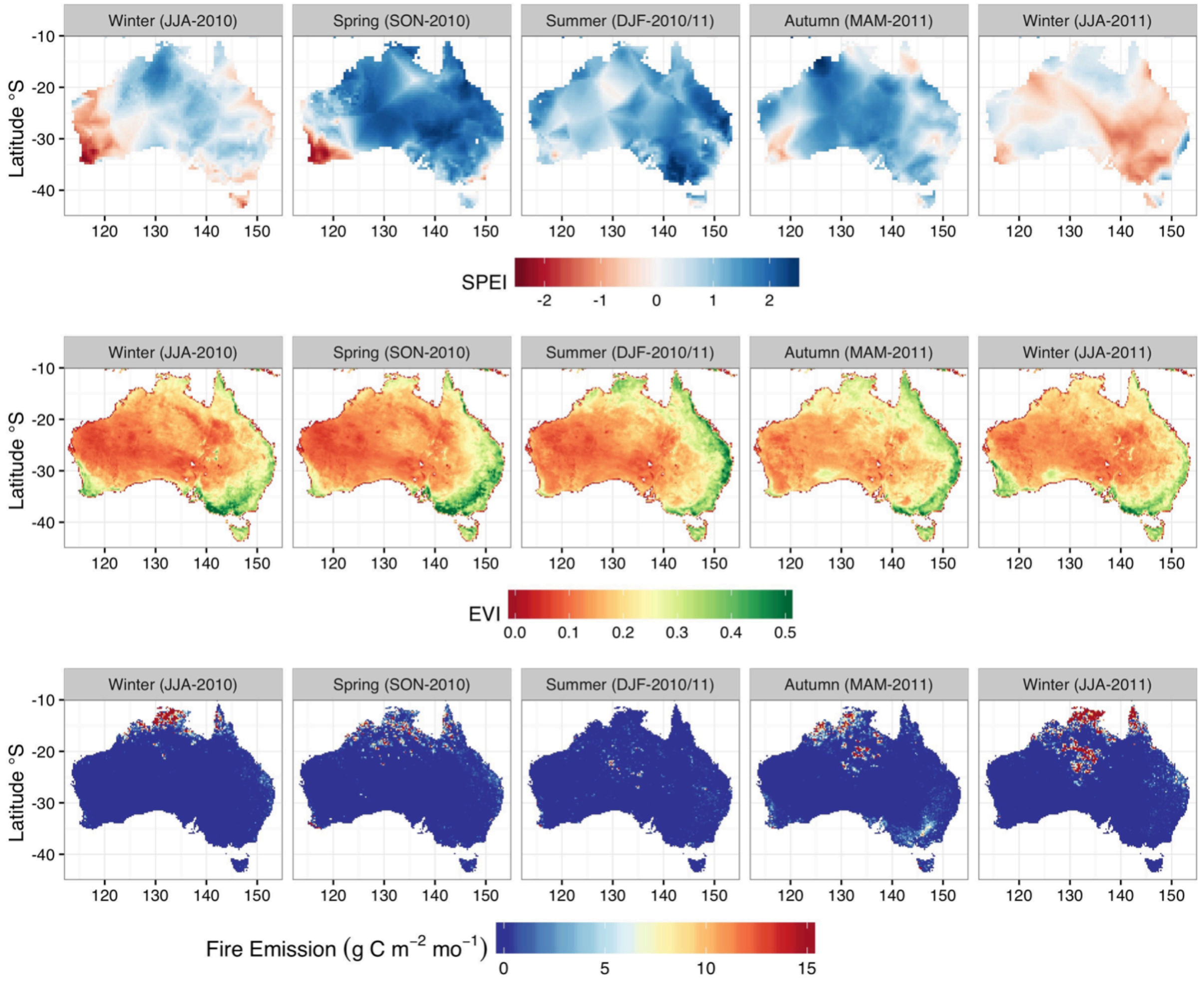

Supplementary Figure S5. Seasonal variations in SPEI, EVI, and fire emission for the 2010-11 wet period. The five panels show seasons from Austral winter (June-August, 2010), spring (September-November, 2010), summer (December-2010 to February-2011), autumn (March-May, 2011), and winter (June-August, 2011). Maps were drawn using R version 3.1.2 (http://www.r-project.org/).

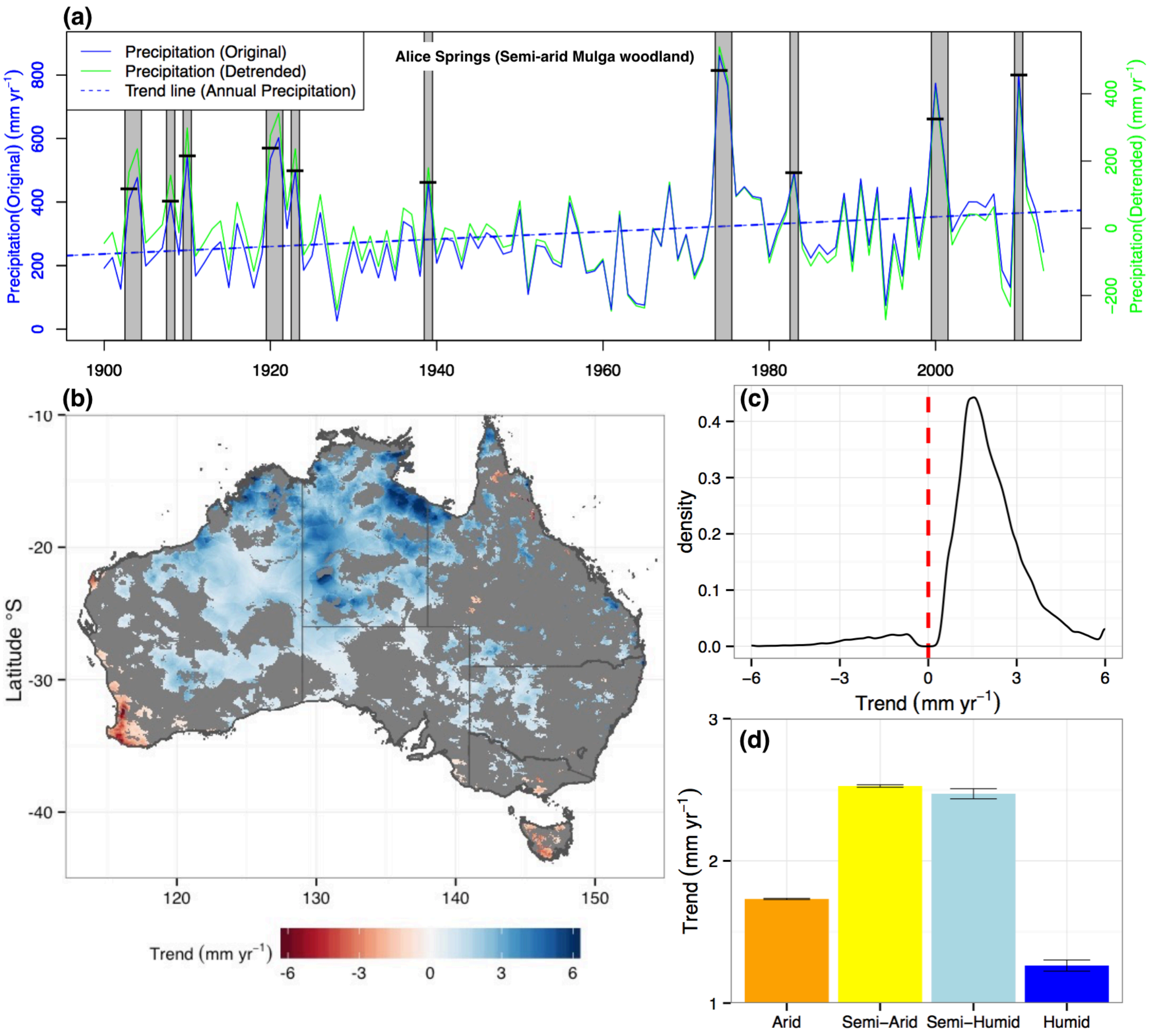

Supplementary Figure S6. Trend in precipitation amounts during the very wet years across Australia from 1900s to 2013. (a) A graphical illustration of the method for identifying very wet years using long-term annual precipitation data from one meteorological station; (b) map of the trend in precipitation amounts during the very wet years from 1900 to 2013 over entire Australia. Grey areas indicate pixels without significant trend ($p < 0.05$) detected; (c) distribution of the trend in precipitation amounts during the very wet years across Australia; (d) zonal average of trend in precipitation amounts during the very wet years by climatic zones over Australia. Map was drawn using R version 3.1.2 (http://www.r-project.org/).

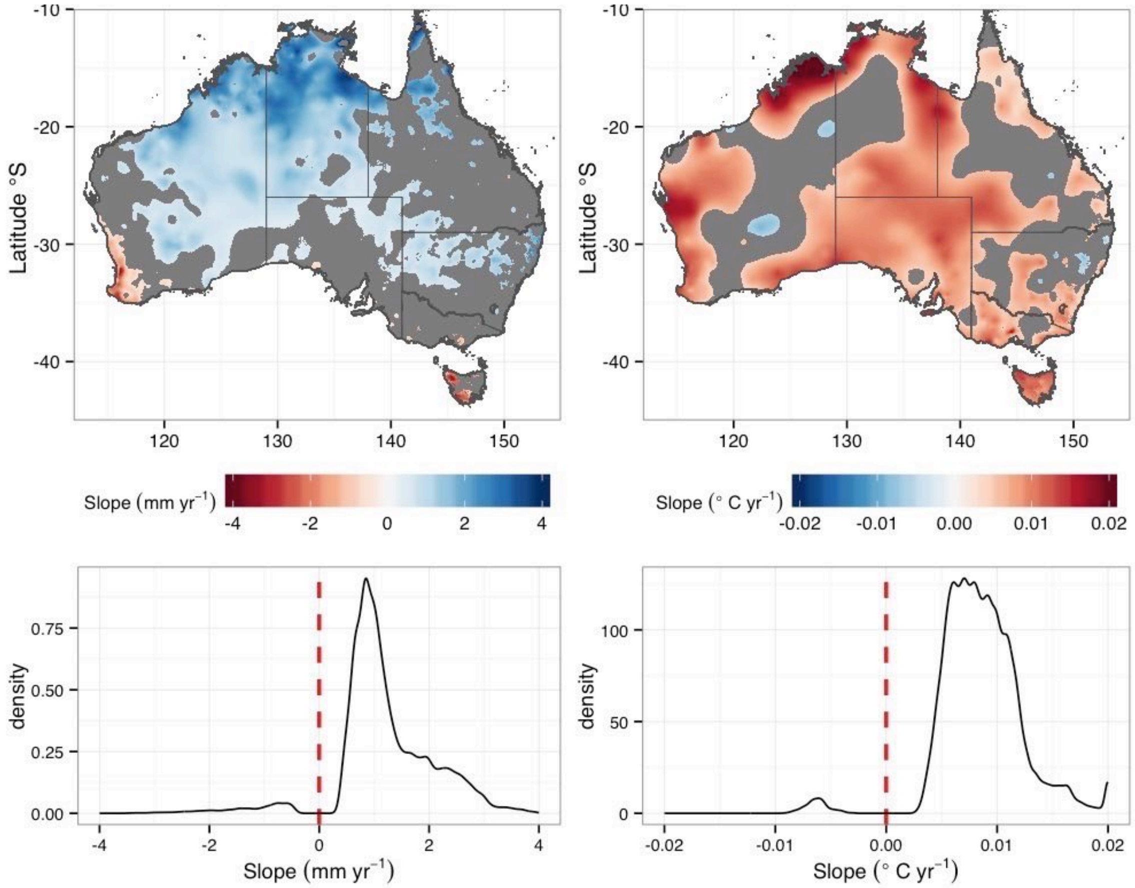

Supplementary Figure S7. Trend in precipitation and temperature across Australia from 1900s to 2013. For both annual precipitation (left) and annual mean daily temperature (right), the trend is expressed as the slope of linear regression model, with the unit of mm yr^{-1} for precipitation and °C yr^{-1} for temperature, respectively. Grey areas indicate the pixels without a significant trend ($p < 0.05$). Below panels show the empirical probability density function plots for significant trend of precipitation ($p < 0.05$) and significant trend of temperature ($p < 0.05$) over Australia respectively. Maps were drawn using R version 3.1.2 (http://www.r-project.org/).

3. Supplementary references

1. Peylin, P. *et al*. Global atmospheric carbon budget: results from an ensemble of atmospheric CO_2-inversions. *Biogeosci* **10**, 6699–6720 (2013).

2. Crisp, D. *et al*. The ACOS CO_2 retrieval algorithm; Part II: Global XCO_2 data characterization. *Atmos Meas Tech* **5**, 687–707 (2012).

3. Houweling, S. *et al*. An intercomparison of inverse models for estimating sources and sinks of CO_2 using GOSAT measurements. *J Geophys Res A* **120**, 5253–5266 (2015).

4. Yokota, T. *et al*. Global Concentrations of CO_2 and CH_4 Retrieved from GOSAT: First Preliminary Results . *Sola* **5**, 160–163 (2009).

5. Chevallier, F. *et al.* CO_2 surface fluxes at grid point scale estimated from a global 21-year reanalysis of atmospheric measurements. *J Geophys Res* **115**, D21307 (2010).

6. Chevallier, F. On the statistical optimality of CO_2 atmospheric inversions assimilating CO_2 column retrievals. *Atmos Chem Phys* **15**, 11133-11145 (2015).

7. Chevallier, F. *et al.* Climate Assessment Report for the GHG-CCI project of ESA's Climate Change Initiative, pp.87, version 2, 22 April 2015.

8. Locatelli, R. *et al.* Atmospheric transport and chemistry of trace gases in LMDz5B: evaluation and implications for inverse modelling. *Geosci Model Dev* **8**, 129–150 (2015).

9. Krinner, G. *et al.* A dynamic global vegetation model for studies of the coupled atmosphere-biosphere system. *Global Biogeochem Cy* **19**, GB1015 (2005).

10. van der Werf, G. R. *et al.* Global fire emissions and the contribution of deforestation, savanna, forest, agricultural, and peat fires (1997–2009). *Atmos Chem Phys* **10**, 11707–11735 (2010).

11. Chevallier, F. *et al.* Contribution of the Orbiting Carbon Observatory to the estimation of CO_2 sources and sinks: Theoretical study in a variational data assimilation framework. *J Geophys Res* **112**, D09307 (2007).

12. Huete, A. R. *et al.* Overview of the radiometric and biophysical performance of the MODIS vegetation indices. *Remote Sens Environ* **83**, 195–213 (2002).

13. Ma, X. *et al.* Parameterization of an ecosystem light-use-efficiency model for predicting savanna GPP using MODIS EVI. *Remote Sens Environ* **154**, 1–19 (2014).

14. Baker, N. R. Chlorophyll Fluorescence: A Probe of Photosynthesis In Vivo. *Annu Rev Plant Biol* **59**, 89–113 (2008).

15. Joiner, J. *et al.* Global monitoring of terrestrial chlorophyll fluorescence from moderate-spectral-resolution near-infrared satellite measurements: methodology, simulations, and application to GOME-2. *Atmos Meas Tech* **6**, 2803–2823 (2013).

16. Guanter, L. *et al.* Global and time-resolved monitoring of crop photosynthesis with chlorophyll fluorescence. *Proc Natl Acad Sci USA* **111**, E1327–33 (2014).

17. Joiner, J. *et al.* The seasonal cycle of satellite chlorophyll observations and its relationship to vegetation phenology and ecosystem-atmosphere carbon exchange. *Remote Sens Environ* **152**, 375-391 (2014).

18. Parazoo, N. C. *et al.* Terrestrial gross primary production inferred from satellite fluorescence and vegetation models. *Glob Change Biol* **20**, 3103–3121 (2014).

19. Yoshida, Y. *et al.* The 2010 Russian drought impact on satellite measurements of solar-induced chlorophyll fluorescence: Insights from modeling and comparisons with parameters derived from satellite reflectances. *Remote Sens Environ* **166**, 163-177 (2015).

20. Eamus, D. *et al.* Carbon and water fluxes in an arid-zone Acacia savanna woodland: An analyses of seasonal patterns and responses to precipitation events. *Agr Forest Meteorol* **182**, 225–238 (2013).

21. Cleverly, J. *et al.* Dynamics of component carbon fluxes in a semi-arid Acacia woodland, central Australia. *J Geophy Res G* **118**, 1168-1185 (2013).

22. Meyer, W. S., Kondrlovà, E. & Koerber, G. R. Evaporation of perennial semi-arid woodland in southeastern Australia is adapted for irregular but common dry periods. *Hydrol Process* **29**, 3714-3726 (2015).

23. Vicente-Serrano, S. M., Beguería, S., López-Moreno, J. I. A multiscalar drought index sensitive to global warming: The standardized precipitation evapotranspiration index. *J Clim* **23**, 1696–1718 (2010).

24. Wahr, J., Swenson, S., Zlotnicki, V. & Velicogna, I. Time-variable gravity from GRACE: First results. *Geophy Res Lett* **31**, L11501–4 (2004).

25. Jones, D. A., Wang, W. & Fawcett, R. High-quality spatial climate data-sets for Australia. *Aust Meteorol Ocean* **58**, 233–248 (2009).

26. Schneider, U., Becker, A., Meyer-Christoffer, A., Ziese, M. & Rudolf, B. Global precipitation analysis products of the GPCC. *Global Precipitation Climatology Centre (GPCC), DWD, Internet*

Publikation, **112** (2008).

27. Giglio, L., Randerson, J. T., & Werf, G. R. Analysis of daily, monthly, and annual burned area using the fourth - generation global fire emissions database (GFED4). *J Geophys Res G*, **118**, 317 - 328 (2013).

28. Randerson, J. T., Chen, Y., van der Werf, G. R., Rogers, B. M., & Morton, D. C. Global burned area and biomass burning emissions from small fires. *J Geophys Res A*, **117**, G04012 (2012).

29. NVIS. Australia's native vegetation - A summary of Australia's major vegetation groups (2007).

30. Huntley, B. J. & Walker, B. H. Ecology of tropical savannas. Vol. 42. Springer-Verlag Berlin-Heidelberg (1982).
